# Supplementary figures and images for: Effects of vitamin D deficiency on blood lipids and bone metabolism: a large cross-sectional study
Source: J Orthop Surg Res. 2023 Jan 7;18:20. doi: 10.1186/s13018-022-03491-w (PMC9826596; doi:10.1186/s13018-022-03491-w)

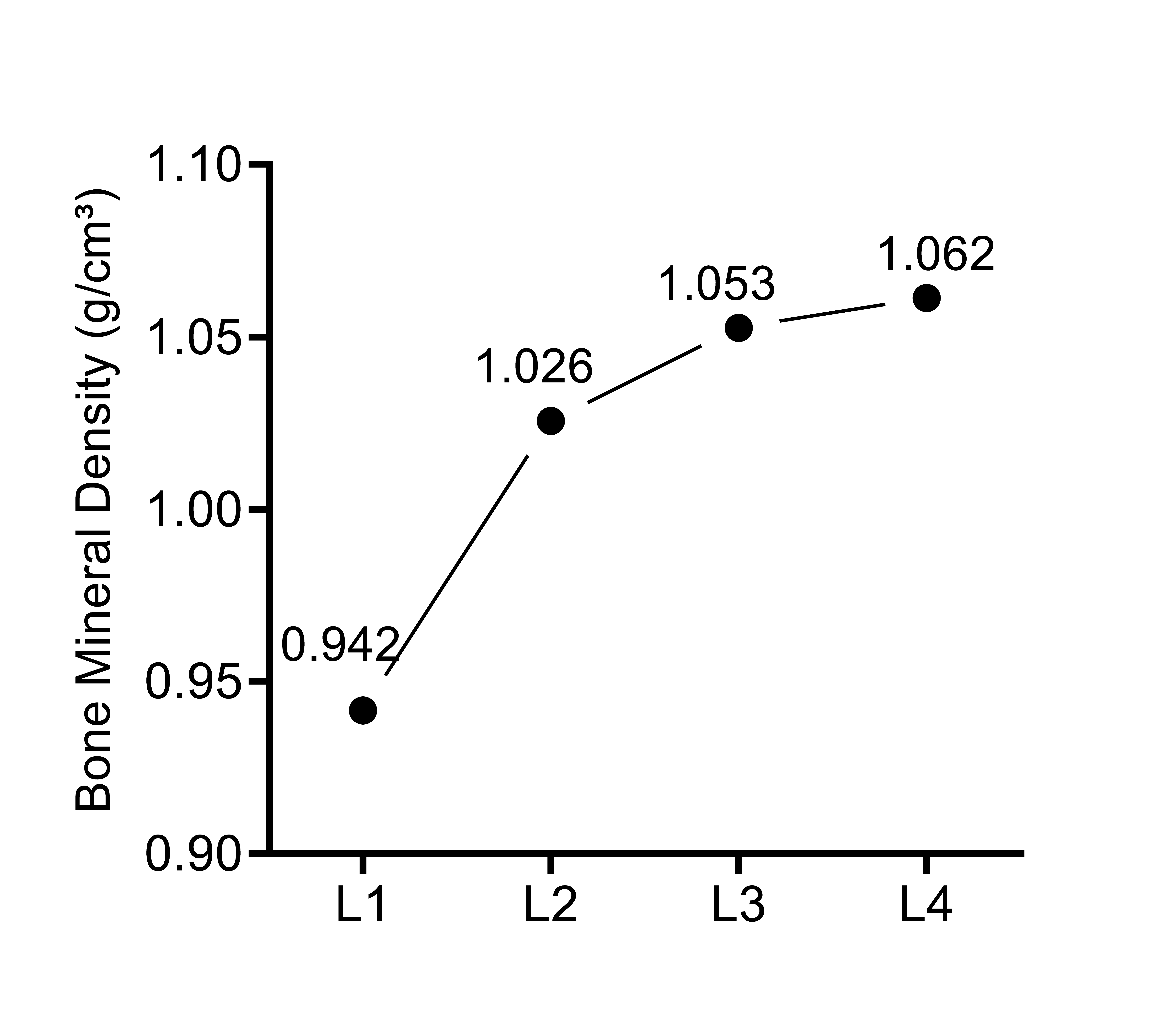

Supplement: Supplementary file 1 — Additional file 1. Figure S1. Bone mineral density of different lumbar vertebrae in participants. [file 13018_2022_3491_MOESM1_ESM.tif]
