# Supplementary material for: Effects of vitamin D deficiency on blood lipids and bone metabolism: a large cross-sectional study
Source: J Orthop Surg Res. 2023 Jan 7;18:20. doi: 10.1186/s13018-022-03491-w (PMC9826596; doi:10.1186/s13018-022-03491-w)
Supplement: Supplementary file 2 — Additional file 2. Table S1. Correlation between high-density lipoprotein cholesterol and bone mineral density based on covariate status classification. [file 13018_2022_3491_MOESM2_ESM.docx]

**Supplementary Table1** Correlation between high-density lipoprotein cholesterol and bone mineral density based on covariate status classification.

| **Covariant** | | **TOTAL.SPINE** | **L1** | **L2** | **L3** | **L4** |
| --- | --- | --- | --- | --- | --- | --- |
|  |  | **β(95%CI)** | **β(95%CI)** | **β(95%CI)** | **β(95%CI)** | **β(95%CI)** |
|  |  | **P** | **P** | **P** | **P** | **P** |
| RACE | Mexican American | -0.010 (-0.040, 0.020) 0.5094 | -0.020 (-0.051, 0.010) 0.1877 | -0.006 (-0.037, 0.025) 0.7073 | -0.005(-0.037,0.028) 0.7672 | -0.011 (-0.043, 0.022)  0.5212 |
|  | Other Hispanic | -0.016 (-0.060, 0.027) 0.4570 | -0.041 (-0.086, 0.003) 0.0692 | -0.013 (-0.057, 0.032) 0.5779 | -0.017 (-0.064, 0.029) 0.4593 | -0.001 (-0.049, 0.046) 0.9517 |
|  | Non-Hispanic White | -0.017 (-0.035, 0.001) 0.0720 | -0.021 (-0.039, -0.003) 0.0234 | -0.017 (-0.036, 0.002) 0.0760 | -0.016 (-0.036, 0.004) 0.1111 | -0.014 (-0.034, 0.005) 0.1548 |
|  | Non-Hispanic Black | -0.041 (-0.069, -0.013) 0.0042 | -0.058 (-0.086, -0.030) <0.0001 | -0.052 (-0.081, -0.023) 0.0004 | -0.035 (-0.065, -0.005) 0.0232 | -0.026 (-0.057, 0.004) 0.0865 |
|  | Other Race | 0.001 (-0.079, 0.080) 0.9881 | -0.003 (-0.084, 0.078) 0.9396 | -0.016 (-0.098, 0.067) 0.7084 | 0.009 (-0.079, 0.096) 0.8487 | 0.007 (-0.077, 0.092) 0.8704 |
| MARITAL | Live with someone | -0.020 (-0.036, -0.005) 0.0102 | -0.025 (-0.040, -0.009) 0.0016 | -0.021 (-0.037, -0.005) 0.0096 | -0.018 (-0.035, -0.001) 0.0350 | -0.019 (-0.036, -0.002) 0.0287 |
|  | Live alone | -0.016 (-0.037, 0.005) 0.1323 | -0.027 (-0.048, -0.005) 0.0145 | -0.021 (-0.043, 0.001) 0.0571 | -0.014 (-0.037, 0.009) 0.2239 | -0.007 (-0.030, 0.015) 0.5284 |
| EDUCATION | Under high school | -0.036 (-0.059, -0.013) 0.0024 | -0.033 (-0.057, -0.010) 0.0050 | -0.035 (-0.058, -0.011) 0.0041 | -0.035 (-0.060, -0.009) 0.0075 | -0.041 (-0.066, -0.016) 0.0014 |
|  | High school or equivalent | -0.023 (-0.050, 0.004) 0.0981 | -0.040 (-0.066, -0.014) 0.0029 | -0.030 (-0.058, -0.002) 0.0381 | -0.013 (-0.043, 0.017) 0.3883 | -0.015 (-0.045, 0.015) 0.3152 |
|  | Some College or AA degree | -0.018 (-0.044, 0.008) 0.1843 | -0.018 (-0.044, 0.009) 0.1928 | -0.019 (-0.046, 0.008) 0.1753 | -0.021 (-0.049, 0.007) 0.1337 | -0.014 (-0.043, 0.015) 0.3374 |
|  | College Graduate or above | 0.005 (-0.021, 0.031) 0.7104 | -0.009 (-0.035, 0.018) 0.5180 | -0.003 (-0.030, 0.025) 0.8579 | 0.008 (-0.020, 0.037) 0.5643 | 0.017 (-0.010, 0.045) 0.2208 |
| SMOKE | Never | -0.020 (-0.047, 0.007) 0.1530 | -0.020 (-0.047, 0.007) 0.1445 | -0.029 (-0.057, -0.002) 0.0376 | -0.013 (-0.042, 0.016) 0.3885 | -0.019 (-0.048, 0.011) 0.2079 |
|  | Former | -0.008 (-0.033, 0.016) 0.5095 | -0.018 (-0.043, 0.006) 0.1370 | -0.010 (-0.035, 0.015) 0.4408 | -0.008 (-0.035, 0.019) 0.5690 | -0.001 (-0.028, 0.026) 0.9600 |
|  | Current | -0.028 (-0.045, -0.010) 0.0018 | -0.034 (-0.051, -0.016) 0.0002 | -0.028 (-0.046, -0.010) 0.0026 | -0.027 (-0.046, -0.008) 0.0057 | -0.025 (-0.044, -0.006) 0.0092 |
| ALP | Q1 | -0.024 (-0.047, -0.001) 0.0439 | -0.035 (-0.058, -0.011) 0.0037 | -0.033 (-0.056, -0.009) 0.0063 | -0.018 (-0.044, 0.007) 0.1577 | -0.014 (-0.039, 0.011) 0.2720 |
|  | Q2 | -0.003 (-0.029, 0.023) 0.8126 | -0.011 (-0.037, 0.016) 0.4388 | -0.003 (-0.030, 0.025) 0.8383 | 0.002 (-0.027, 0.030) 0.9060 | -0.002 (-0.031, 0.026) 0.8683 |
|  | Q3 | -0.016 (-0.043, 0.010) 0.2337 | -0.023 (-0.050, 0.003) 0.0852 | -0.012 (-0.040, 0.016) 0.4037 | -0.014 (-0.043, 0.015) 0.3326 | -0.019 (-0.048, 0.011) 0.2114 |
|  | Q4 | -0.044 (-0.069, -0.018) 0.0008 | -0.045 (-0.070, -0.019) 0.0006 | -0.049 (-0.076, -0.022) 0.0003 | -0.047 (-0.076, -0.019) 0.0010 | -0.036 (-0.064, -0.009) 0.0095 |

Age, sex, Had at least 12 alcohol drinks past one year?, Hypertension, Diabetes, Total 25(OH)D, BMI (obese, overweight, normal), TC(quartile groups), Ca (quartile groups), P (quartile groups), ALT (quartile groups), AST (quartile groups), CRP (quartile groups) were adjusted in the model
